# Targeting OCT2 with Duloxetine to Prevent Oxaliplatin-induced Peripheral Neurotoxicity

**DOI:** 10.1158/2767-9764.CRC-22-0172

**Published:** 2022-11-03

**Authors:** Mahesh R. Nepal, Hanieh Taheri, Yang Li, Zahra Talebi, Muhammad Erfan Uddin, Yan Jin, Duncan F. DiGiacomo, Alice A. Gibson, Maryam B. Lustberg, Shuiying Hu, Alex Sparreboom

**Affiliations:** 1 Division of Pharmaceutics and Pharmacology, College of Pharmacy and Comprehensive Cancer Center, The Ohio State University, Columbus, Ohio.; 2 Division of Outcomes and Translational Sciences, College of Pharmacy and Comprehensive Cancer Center, The Ohio State University, Columbus, Ohio.; 3 The Breast Center at Smilow Cancer Hospital at Yale, Yale School of Medicine, New Haven, Connecticut.

## Abstract

Oxaliplatin-induced peripheral neurotoxicity (OIPN) is a debilitating side effect that afflicts approximately 90% of patients that is initiated by OCT2-dependent uptake of oxaliplatin in dorsal root ganglion (DRG) neurons. The antidepressant drug duloxetine has been used to treat OIPN, although its usefulness in preventing this side effect remains unclear. We hypothesized that duloxetine has OCT2-inhibitory properties and can be used as an adjunct to oxaliplatin-based regimens to prevent OIPN. Transport studies were performed in cells stably transfected with mouse or human OCT2 and in isolated mouse DRG neurons *ex vivo*. Wild-type and OCT2-deficient mice were used to assess effects of duloxetine on hallmarks of OIPN, endogenous OCT2 biomarkers, and the pharmacokinetics of oxaliplatin, and the translational feasibility of a duloxetine-oxaliplatin combination was evaluated in various models of colorectal cancer. We found that duloxetine potently inhibited the OCT2-mediated transport of several xenobiotic substrates, including oxaliplatin, in a reversible, concentration-dependent manner, and independent of species and cell context. Furthermore, duloxetine restricted access of these substrates to DRG neurons *ex vivo* and prevented OIPN in wild-type mice to a degree similar to the complete protection observed in OCT2-deficient mice, without affecting the plasma levels of oxaliplatin. Importantly, the uptake and cytotoxicity of oxaliplatin in tumor cell lines *in vitro* and *in vivo* were not negatively influenced by duloxetine. The observed OCT2-targeting properties of duloxetine, combined with the potential for clinical translation, provide support for its further exploration as a therapeutic candidate for studies aimed at preventing OIPN in patients with cancer requiring treatment with oxaliplatin.

**Significance:**

We found that duloxetine has potent OCT2-inhibitory properties and can diminish excessive accumulation of oxaliplatin into DRG neurons. In addition, pretreatment of mice with duloxetine prevented OIPN without significantly altering the plasma pharmacokinetics and antitumor properties of oxaliplatin. These results suggest that intentional inhibition of OCT2-mediated transport by duloxetine can be employed as a prevention strategy to ameliorate OIPN without compromising the effectiveness of oxaliplatin-based treatment.

## Introduction

Oxaliplatin is a third-generation platinum-based chemotherapeutic agent used primarily to treat advanced cases of colorectal cancer. Oxaliplatin-induced peripheral neurotoxicity (OIPN) is a debilitating, dose-limiting side effect of oxaliplatin that affects up to 90% of patients with cancer (1). Clinical manifestations of OIPN include mechanical allodynia, hyperalgesia, dysesthesia, and paranesthesia and these events can persist for many years and cause chronic disabilities even after discontinuation of treatment (2). Although the exact mechanism by which oxaliplatin causes OIPN remains unclear, it has been suggested that the side effect is initiated by oxaliplatin uptake into satellite glial cells (SGC) of dorsal root ganglion (DRG) neurons, a process that is mediated by the organic cation transporter OCT2 (SLC22A2; refs. 3, 4). The notion that OIPN is dependent on uptake transport by OCT2 has led to the thesis that pharmacologic inhibition of this transport mechanism may present a strategy to diminish excessive accumulation of oxaliplatin in the target cells within the peripheral nervous system and thereby prevent downstream events that ultimately result in OIPN (3, 5). Proof-of-concept studies have suggested that this is a feasible therapeutic strategy with translational potential, and this recognition has triggered several studies aimed at identifying novel potent OCT2 inhibitors (6). In our previously reported small-molecule library screen of >8,000 bioactive compounds, the selective serotonin-norepinephrine reuptake inhibitor duloxetine (Supplementary Fig. S1) was identified as a potential inhibitor of OCT2 (ref. 5; Supplementary Table S1). Interestingly, several preclinical and clinical studies have shown that duloxetine can partially reverse the neuropathic pain associated with oxaliplatin (7, 8), vincristine (9, 10), and paclitaxel (11), and duloxetine is currently the only American Society of Clinical Oncology– and European Society for Medical Oncology (ESMO)-recommended treatment of OIPN (11–13). We here explored the hypotheses that duloxetine is a potent inhibitor of OCT2 *in vitro* and that pretreatment with duloxetine *in vivo* can inhibit the entry of oxaliplatin into DRG neurons by blocking OCT2 function, thereby preventing OIPN without influencing the pharmacokinetic and antitumor properties of oxaliplatin.

## Materials and Methods

### Cellular Accumulation

Cellular uptake studies were performed in the presence or absence of varying concentrations of duloxetine in HEK293 and Hela cells (ATCC) engineered to overexpress mouse (m), rat (r), or human (h) orthologs of the organic anion transporting polypeptides hOATP1B1, hOATP1B3, and mOATP1B2, the organic cation transporters hOCT1, hOCT2, mOCT2, rOCT2, hOCT3, and the multidrug and toxin extrusion proteins mMATE1 and hMATE1. Transport function was evaluated using radiolabeled prototypical substrates (Supplementary Table S2), including estradiol [6,7-^3^H(N)]-17β-D-glucuronide (specific activity, 50 Ci/mmol; purity, 99%; American Radiolabeled Chemicals; EβG) for OATP1B1 and OATP1B2, the cholecystokinin octapeptide [proprionyl-^3^H(N)]-CCK-8 (specific activity, 115 Ci/mmol; purity, >90%; Perkin Elmer; CCK-8) for OATP1B3, [ethyl-1-^14^C]-tetraetylammonium chloride (specific activity, 55 mCi/mmol; purity, >99%; American Radiolabeled Chemicals; TEA) for OCT1, rOCT2, and MATE1, [biguanidine-^14^C]-metformin hydrochloride (specific activity, 112 mCi/mmol; purity, 100%; Moravek) for OCT3, and TEA, [cyclohexane ring-^14^C]-oxaliplatin (specific activity, 54 mCi/mmol, purity, 98%; Moravek), and the fluorescent substrate 4-(4-(dimethylamino)styryl)-N-methylpyridinium iodide (purity, >97%; Sigma-Aldrich; ASP) for mOCT2 and hOCT2. The influence of the duloxetine metabolites 5-hydroxy-6-methoxy-duloxetine and 4-hydroxy-duloxetine-glucuronide (Cayman Chemical) on the function of mOCT2 and hOCT2 was evaluated using ASP as the substrate.

Hela and HEK-293 cells were procured from ATCC in 2013 and 2015, respectively. Cells were authenticated by short tandem repeat profiling (OSUCCC Genomics Shared Resource) and using the Cellosaurus database (Expasy). Colorectal cancer cells were purchased from NCI Frederick Cancer Tumor/Cell Line Repository in 2018. Cell lines were authenticated by Applied Biosystems AmpFISTR Identifiler testing with PCR amplification. Cells were grown in an incubator supplied with 5% CO_2_ that was maintained at 37°C with 95% relative humidity (RH) in RPMI (Thermo Fisher Scientific) with 10% FBS. All the cells were used within passages 30 and verified to be *Mycoplasma* free using the MycoAlert Mycoplasma Detection Kit (Lonza). Cells were grown to confluence (80%–90%) and 0.5 million cells were seeded in polylysine precoated 12-well plates. During uptake study, cells were briefly washed with prewarmed PBS solution (pH 7.4) and pre-incubated with either vehicle or duloxetine prepared in serum-free and phenol red–free DMEM for 15 minutes. After preincubation, media from the cells was removed and cells were treated for 5–30 minutes with respective substrates in presence or absence of duloxetine. Intracellular radioactivity originating from the radiolabeled substrates was measured using liquid scintillation counting and intracellular fluorescence originating from ASP was measured by fluorimetry. For washout assays, cells were preincubated with duloxetine for 15 minutes, media containing duloxetine was removed and cells were incubated for 15 minutes with TEA at 0, 15, 30, and 180 minutes after the removal of duloxetine. Following incubation, uptake was stopped by washing cells three times with ice-cold PBS buffer and uptake of TEA was measured by a scintillation counting. Uptake results were normalized to total protein content and expressed as percentage of results obtained in cells transfected with empty vector controls. Competitive counterflow assays (CCF) were conducted with TEA, a known substrate of OCT2 according to published procedures, with minor modifications (14). In brief, cells overexpressing OCT2, or control cells were seeded in 24-well plates with 10% FBS for 24 hours. On the day of the experiment, medium from cells was removed and cells were washed with prewarmed CCF buffer composed of NaCl: 135 mmol/L, HEPES: 13 mmol/L, CaCl_2_·2H2O: 2.5 mmol/L, MgCl_2_: 1.2 mmol/L, MgSO_4_·7H_2_O: 0.8 mmol/L, KCl: 5 mmol/L, and D-glucose: 28 mmol/L, with the pH adjusted to 7.4. The cells were then incubated with 2 μmol/L TEA for 3 minutes at 37°C and further incubated with TEA with or without duloxetine (200 μmol/L) for 1 minute. Immediately after incubation, medium from cells was removed, and reaction was terminated by washing cells three times with cold PBS. Metformin was used as a positive control substrate and dasatinib was used as a negative control substrate in the experiments. All cellular uptake assays were carried out independently by two different investigators on multiple separate occasions.

### Animal Models

Wild-type mice and mice deficient for OCT1/OCT2, the murine orthologs of human OCT2, on an FVB background [OCT1/2(−/−) mice] were obtained from Taconic Biosciences and were bred in-house at The Ohio State University. Athymic nude mice (CrTac:NCr-Fox1nu; NCRNU-M) were also obtained from Taconic Biosciences. Previous investigations indicated that the systemic clearance, tissue distribution, and excretion of oxaliplatin is similar in male and female mice (4), and that oxaliplatin-induced mechanical allodynia in mice does not exhibit sexual dimorphism (3). Because sex-dependent effects were not anticipated in the present studies, all experiments were performed only in male mice. All animals were housed in a controlled environment with a 12-hour light-dark cycle, provided with food and water *ad libitum* and handled according to the Animal Care and Use Committee of The Ohio State University, under an approved protocol 2015A00000101-R2. All animals purchased from external vendors were acclimatized for at least 1 week before starting the experiment. At least 5 animals were used for each test groups unless otherwise specified. Mice were balanced among groups in terms of group size and baseline characteristics such as weight, sex, and age, followed by the random assignment to control and intervention groups, according to procedures outlined elsewhere (15).

### Isolation of DRGs and SGCs

DRG neurons from mice treated with oxaliplatin or the duloxetine-oxaliplatin combination were obtained from thoracic position 8 (T_8_) to lumbar position 5 (L_5_) and total platinum levels originating from oxaliplatin were analyzed by a validated method based on flameless atomic absorption spectrometry (4). For cellular uptake assays of OCT2 substrates, satellite cells were isolated as described previously (3). In brief, DRG from wild-type mice and OCT1/2(−/−) mice were collected in PBS without Ca^2+^ and Mg^2+^, supplemented with d-glucose and the antibiotics penicillin and streptomycin (Gibco). Next, DRG were digested with type II collagenase solution for 60 minutes followed by digestion with trypsin for 10 minutes. The effect of trypsin was neutralized with the addition of full DMEM containing 1% penicillin/streptomycin. Single satellite cells were prepared by pipetting up and down with a 1 mL pipette for several times, and digested DRG were transferred to a 25 cm^2^ flask and incubated for additional 3 hours in a 37°C incubator with 5% CO_2_ and 95% RH. Suspended neuronal debris was removed and SGCs were further cultured until confluency. Uptake studies were performed as described above for HEK293 and Hela cells.

### Evaluation of Peripheral Neurotoxicity

Mechanical allodynia in mice was used as an initial readout of drug-induced peripheral neurotoxicity using a Von Frey Hair (VFH) test as described previously (3). In brief, for acute neurotoxicity testing, a single dose of 10 mg/kg oxaliplatin (dissolved in 5% glucose solution), 1 mg/kg vincristine (dissolved in 0.9% normal saline), and 10 mg/kg paclitaxel [formulated in cremophor EL/ethanol (1:1, v/v), diluted in 0.9% normal saline] was administered by intraperitoneal or intravenous injection. For chronic neurotoxicity testing, multiple doses of oxaliplatin (4 mg/kg dissolved in 5% glucose solution or vehicle) were administered by intraperitoneal injection twice a week for a total of 3 weeks. To determine the neuroprotective effect of duloxetine, duloxetine (30 mg/kg dissolved in 0.9% NaCl solution) or vehicle was administered by intraperitoneal injection to mice 60 minutes before the administration of oxaliplatin, vincristine, or paclitaxel. The *in vivo* doses of oxaliplatin, vincristine, and paclitaxel were chosen based on previously published studies (3, 16, 17). The VFH tests were employed before treatment to establish baseline levels of sensitivity and 24 hours after the administration of oxaliplatin, vincristine, or paclitaxel for acute toxicity testing, and at 24 hours after every second dose of weekly oxaliplatin treatment for the chronic testing. All animals were allowed to acclimatize for 1 hour in a top wire mesh prior to sensitivity testing. Paw withdrawal force in g was expressed as a percentage change from baseline values to normalize interday variability of the results. The analysts involved in drug administration and VFH test evaluation were double blinded to the treatment groups and mouse genotypes.

For nerve conduction studies, a clinical electrodiagnostic system (Ultra Pro S100, Natus Neurology) was used. Supramaximal action potential amplitude and nerve conduction velocity of caudal and sciatic nerves were measured before after completion of treatments. Two recording electrodes were implanted 10 mm proximally, near the ankle, and stimulating electrodes were placed on the fourth digit of the hind paw. Likewise, two recording electrodes were implanted at the base of the tail and two recording electrodes were placed 35 mm proximally. Ten supramaximal stimulations were stimulated to both the sciatic and caudal nerves. The distance between stimulating electrodes to that of distant latency was used to calculate the velocity and peak-to-peak measurement was taken for amplitude. Mice were held under isoflurane anesthesia during the course of nerve conduction testing and heating pads were used to maintain the animals at a constant body temperature.

### Pharmacokinetic Studies

To determine the plasma–time concentration profile of oxaliplatin following drug administration in the presence of absence of pretreatment with duloxetine, a serial blood sampling strategy was employed in which samples (∼25 μL) were collected at 0.25, 0.5, 1, 2, and 4 hours (18). To avoid distress to animals from the repeated withdrawn of blood from the same site, samples were collected from the submandibular vein of mice for first two timepoints, the retroorbital venous plexus in the next two timepoints, and by cardiac puncture for the last timepoint. Plasma samples were obtained by centrifuging blood samples at 11,000 rpm for 5 minutes and storing the supernatant immediately at −80°C until further analysis. Samples were mixed with 0.2% nitric acid solution, vortex mixed, and total platinum levels originating from oxaliplatin in the samples were measured by flameless atomic absorption spectrometry (19). The ability of duloxetine to distribute to DRG neurons in mice was evaluated by intravenous injection of a 20 mg/kg dose containing a tracer of [*G*-^3^H]-duloxetine (specific activity, 0.2 Ci/mmol; purity, 96%; Moravek) in wild-type mice and OCT1/2(−/−) mice and by measuring total radioactivity using liquid scintillation counting. DRG samples were collected 15 minutes after intravenous dosing, and DRG-to-plasma ratios were calculated to estimate the extent of tissue distribution. The influence of duloxetine administration (intraperitoneal injection at 30 mg/kg) on the plasma levels of the OCT2 biomarkers creatinine (20) and 1-N-methylnicotinamide (NMN; ref. 21) was examined in samples taken at 0, 15, 30, 60, and 240 minutes after duloxetine treatment. Levels of creatinine and NMN were measured by a validated method based on LC/MS-MS detection (21). Pharmacokinetic parameters were calculated by noncompartmental analysis using the Phoenix WinNonlin 8.1 software (Certara).

### Cell Viability Assays

The colorectal cancer cells COLO205, KM12, SW620, HCT116, HT15, HT29, and HCC2998 (NCI Frederick Cancer Tumor/Cell Line Repository) were tested to evaluate the influence of duloxetine concentrations of 1 or 10 μmol/L on oxaliplatin uptake or oxaliplatin-induced cell growth inhibition using 3-(4,5-dimethylthiazol-2-yl)-2,5-diphenyltetrazolium bromide) tetrazolium (MTT) assays (Roche Diagnostics) in two-dimensional culture, as described previously (3). Cells were used within 30 passages after thawing and were routinely checked to ensure there was no *Mycoplasma* contamination (MycoAlert Detection Kit). The selection of chosen duloxetine concentrations was derived from a study in which a 30 mg/kg dose of duloxetine was associated with average plasma levels in mice of approximately 6 μmol/L (22). The MTT assays were performed in 96-well plates using cells seeded at a density of 5,000 cells/well. After 24-hour incubation periods, cells were treated with nine different concentrations of either oxaliplatin, duloxetine, or the combination of oxaliplatin and duloxetine for 72 hours in a 37°C incubator supplied with 5% CO_2_ and 95% RH. Following the incubation period, 10 μL of MTT solution (5 mg/mL) was added and incubated for another 4 hours. Formazan crystals were dissolved in 100 μL of 10% SDS and 0.01 mol/L HCl solution and absorbance was measured at 565 nm. All results were presented as the percentage of vehicle controls in the absence of drug. All cell viability assays were carried out independently by two analysts on separate occasions.

### Assessment of *In Vivo* Antitumor Efficacy

Tumor xenograft studies were performed as described previously (3) with minor modifications. In brief, a lentiviral vector pCDH-EF1a-128 eFFly-eGFP (Addgene) and packaging plasmids psPAX and pMD2.G were cotransfected into HEK293T cells. After 48 hours, the viruses were collected and inoculated into HCT116 colon cancer cells, which were then sorted by GFP for luciferase positivity (kind gift from Dr. Jing Wang, OSU). Following expansion, 2 million cells per 100 μL were injected in both right and left flanks of male athymic nude mice. Mice were randomized into treatment groups once tumors had grown to a size of about 100 mm^3^ following measurement of tumor volume by a digital caliper and IVIS imaging such that the average tumor burden at the start of treatment was similar between each group (Supplementary Fig. S2). A digital caliper was used to measure the mice tumor volume with the formula: *V* = *W*^2^ × *L*/2, where *L* is the maximum diameter of the tumor and *W* is the perpendicular diameter. For treatment, mice received twice weekly either vehicle alone, oxaliplatin alone (4 mg/kg per dose) or oxaliplatin (4 mg/kg) given 1 hour after duloxetine (30 mg/kg). The dose of oxaliplatin was selected on the basis of our previous study (3), which corresponds to a total weekly dose of approximately 8 mg/kg and total dose of 24 mg/kg. All treatments were administered by intraperitoneal injections twice per week for a total of 3 weeks. Tumor volume was measured by digital calipers twice weekly and before the start of treatment by bioluminescence imaging (IVIS Lumina I).

### Statistical Analysis

All data are presented as mean ± SEM of replicate observations, and experiments were repeated at least two separate occasions, unless stated otherwise. Group comparisons were done using a Student *t* test (comparisons made between two groups) or one-way ANOVA with a Dunnett *post hoc* test (comparisons made between more than two groups), and *P* < 0.05 was considered statistically significant. Statistical analyses were performed using the software package Prism 9 (GraphPad).

### Data Availability

The data generated in this study are available upon request from the corresponding authors.

## Results

### Inhibition of OCT2 by Duloxetine *In Vitro*

**FIGURE 1:**
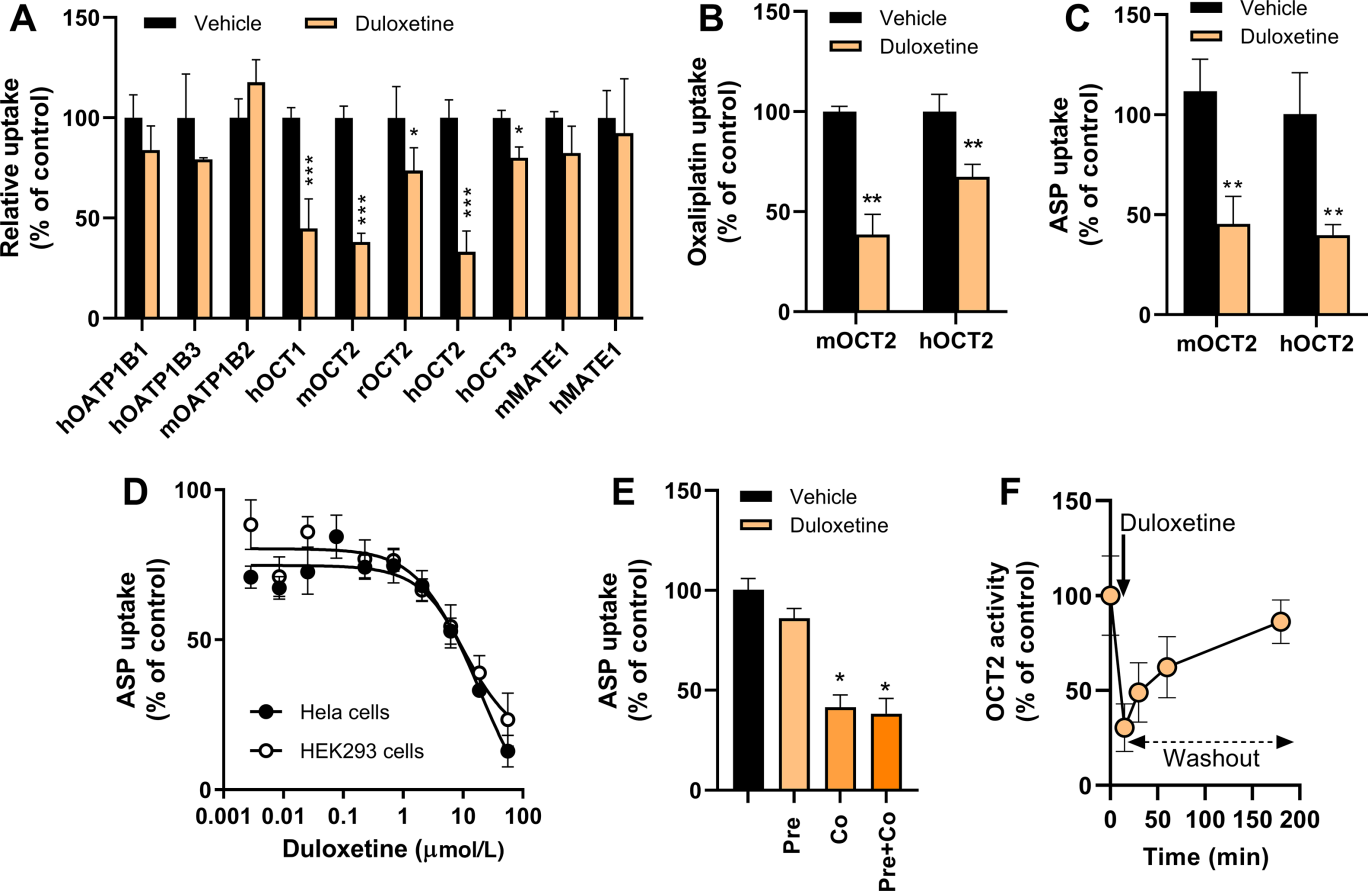
Duloxetine reversibly inhibits the OCT2-dependent transport of multiple substrates. **A,** HEK293 cells overexpressing various mammalian transporters were incubated with prototypical substrate alone or following pretreatment with duloxetine. **B,** Influence of duloxetine on oxaliplatin transport by mouse (m) and human (h) OCT2. **C,** Influence of duloxetine on ASP transport by mOCT2 and hOCT2. **D,** Concentration-dependent inhibition of OCT2-dependent ASP transport by duloxetine in HEK293 and Hela cells overexpressing hOCT2. The concentrations associated with 50% inhibition of OCT2 function were 7.6 μmol/L (HEK293 cells) and 3.98 μmol/L (Hela cells). **E,** Influence of incubation condition on duloxetine-mediated inhibition of OCT2. HEK293 cells overexpressing hOCT2 were either preincubated (duloxetine before oxaliplatin), coincubated (simultaneous duloxetine and oxaliplatin), or preincubated and coincubated (duloxetine before simultaneous duloxetine and oxaliplatin). **F,** Reversibility of duloxetine-mediated inhibition of OCT2. HEK293 cells overexpressing mOCT2 were preincubated with duloxetine for 15 minutes followed by washout of duloxetine and analysis of residual OCT2-mediated transport of TEA. Uptake of each substrate was normalized to total protein content and expressed as percentage of control. Each bar or symbol represents the mean ± SEM (*n* = 3–6 observations per group). *, *P* < 0.05; **, *P* < 0.01; ***, *P* < 0.001 versus corresponding vehicle control.

Our previous identification of duloxetine as a putative OCT2 inhibitor from a small-molecule library screen (5) provided the incentive to examine the influence of duloxetine on the function of OCT2 and various other xenobiotic uptake transporters in engineered cell-based models. These studies confirmed that duloxetine inhibits the transport of TEA by mouse, rat, and human OCT2. Similar inhibitory effects were observed against the structurally related hepatic transporter OCT1, although duloxetine had less profound to no effect on the function of the organic cation transporters OCT3 and MATE1 or on the hepatic organic anion transporting polypeptides OATP1B1 and OATP1B3 (Fig. 1A). Because OCT1 and OCT3 were previously found to not directly contribute to OIPN (3), we focused on OCT2 as a target for duloxetine in subsequent studies.

The OCT2-inhibitory properties of duloxetine were not restricted to TEA, because the same agent also blocked the OCT2-mediated transport of other xenobiotic substrates such as oxaliplatin (Fig. 1B) and ASP (Fig. 1C). Interestingly, the major duloxetine metabolites 5-hydroxy-6-methoxy-duloxetine and 4-hydroxy-duloxetine-glucuronide (23) lacked OCT2-inhibitory properties (Supplementary Fig. S3). Dose–response experiments confirmed that duloxetine-mediated OCT2 inhibition was concentration dependent with no noticeable cell context (Fig. 1D) or species dependence (Supplementary Fig. S3), and that preincubation of cells with duloxetine was not required to observe OCT2 inhibition (Fig. 1E). Moreover, washout experiments indicated that the effect of duloxetine on OCT2 was reversible with complete restoration of function within 3 hours following removal of duloxetine from the cells (Fig. 1F). These observations suggest that duloxetine inhibits the function of OCT2 in a manner that is independent of the substrate, cell context, or species, and that this property is dependent on the concentration of duloxetine and rapidly reversible.

### Duloxetine Blocks the Uptake of Oxaliplatin into DRG Neurons

**FIGURE 2:**
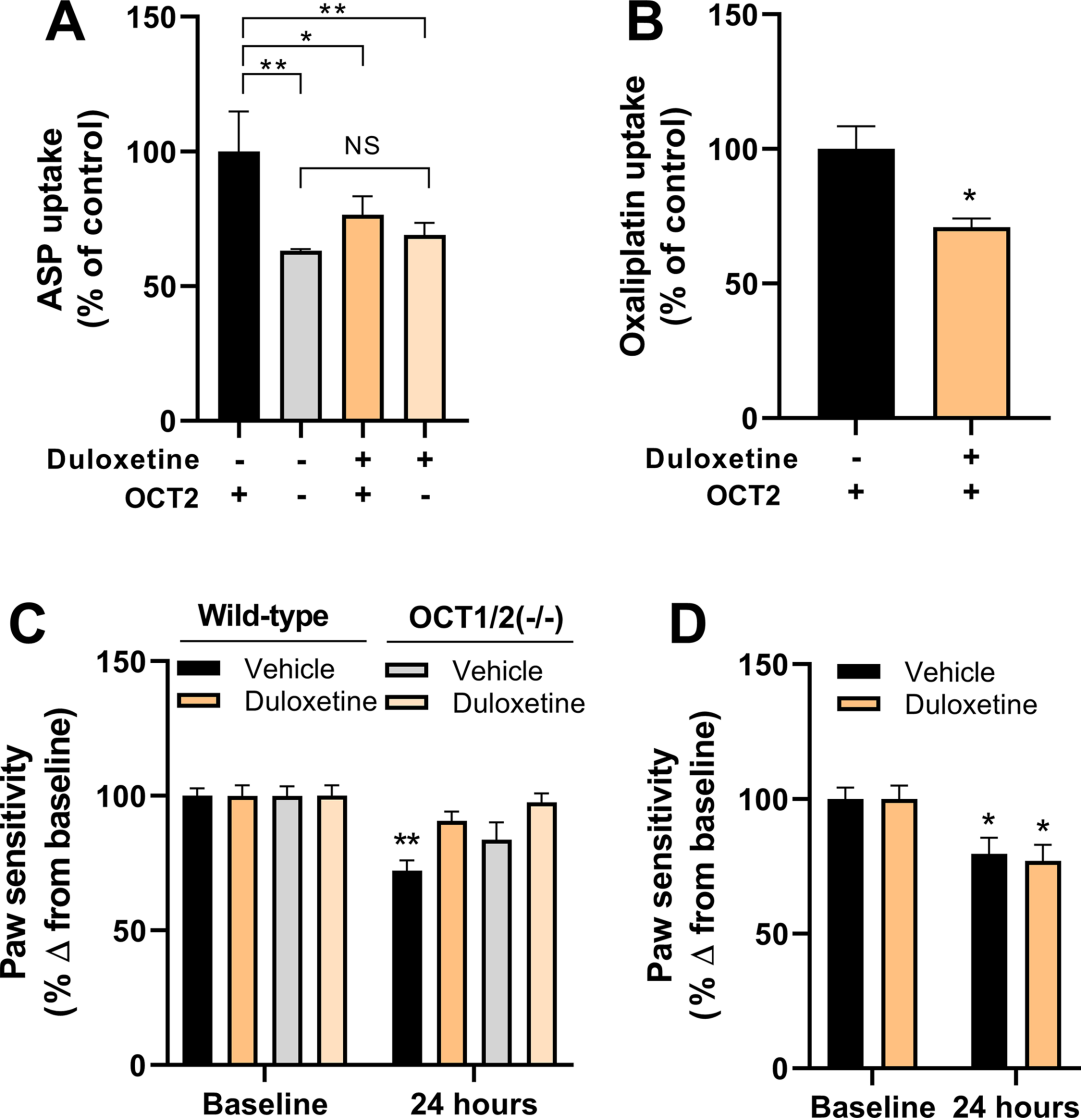
Duloxetine protects against OIPN by blocking oxaliplatin entry to DRG neurons. **A,** Effect of duloxetine on the transport of ASP in SGCs isolated from wild-type mice and OCT1/2(−/−) mice (*n* = 3 per group). **B,** Effect of duloxetine on the transport of oxaliplatin in SGCs isolated from wild-type mice (*n* = 3 per group). **C,** Influence of duloxetine administered 1 hour before oxaliplatin on mechanical allodynia in wild-type mice and OCT1/2(−/−) mice measured by VFH tests (*n* = 10 per group). **D,** Influence of duloxetine administered 1 hour after oxaliplatin on mechanical allodynia in wild-type mice measured by VFH tests (*n* = 5 per group). Data represent the percentage change from baseline and each bar represents the mean ± SEM of two independent experiments. *, *P* < 0.05; **, *P* < 0.01 versus corresponding control or baseline.

We previously reported that OIPN is dependent on OCT2-mediated uptake of oxaliplatin into SGCs within DRG neurons, and that this process is sensitive to genetic or pharmacologic knockout of OCT2 (3). To evaluate the ability of duloxetine to affect this neuronal transport process, we initially performed *ex vivo* uptake studies with ASP in SGC isolated from wild-type mice and OCT1/2(−/−) mice. Similar to observations made in engineered cells, ASP was taken up in OCT2-proficient cells and uptake was significantly impaired under OCT2-deficient conditions (Fig. 2A). Pretreatment with duloxetine inhibited the uptake of ASP to a degree similar to that observed in OCT1/2(−/−) cells in the absence of duloxetine (*P* = 0.02), whereas duloxetine did not further influence the uptake of ASP in SGC from OCT1/2(−/−) mice (*P* = 0.08; Fig. 2A). Similar observations were made using oxaliplatin as the test substrate in SGCs isolated from wild-type mice (Fig. 2B), suggesting that duloxetine can restrict the access of oxaliplatin to DRG neurons in an OCT2-dependent manner.

To evaluate the feasibility of duloxetine to modulate the function of neuronal OCT2 function *in vivo*, we next verified that high levels of duloxetine (∼3 μg/g) were present in DRG neurons 15 minutes after the intravenous administration of duloxetine containing a radiotracer, with an estimated DRG-to-plasma concentration ratio of 4.35 ± 0.06 in wild-type mice and 4.99 ± 0.13 in OCT1/2(−/−) mice (Supplementary Fig. S4). This suggests that duloxetine has easy access to the peripheral nervous system at the exact site associated with OIPN regardless of OCT1/2-genotype status. To test whether duloxetine is itself a transported substrate of OCT2, we initially evaluated the direct uptake of radiolabeled duloxetine in HEK293 cells overexpressing mouse OCT2 or HEK293 or Hela cells overexpressing human OCT2. In these experiments, we observed a high background reading in both the empty vector control and OCT2-overexpressed cells (Supplementary Fig. S4), presumably due to excessive nonspecific binding of duloxetine to extracellular proteins on the outer membrane. Regardless, the observed differences between control cells and cells overexpressing OCT2 were less than 2-fold, suggesting duloxetine might not be an OCT2 substrate as per the FDA guidance (24). To confirm this hypothesis, we next performed a competitive counterflow assay using TEA as the test substrate. In this assay, the positive control substrate metformin affected intracellular levels of TEA, whereas dasatinib, a negative control substrate, or duloxetine, even at a concentration of 200 μmol/L, did not (Supplementary Fig. S4). This suggests that duloxetine transport likely occurs independently of OCT2.

Next, we evaluated the hypothesis that pretreatment with duloxetine would prevent OIPN by performing a VFH test to assess mechanical allodynia. As predicted from our *in vitro* and *ex vivo* studies, duloxetine administered 1 hour before oxaliplatin protected against OIPN (Fig. 2C), whereas treatment of wild-type mice with duloxetine 1 hour after oxaliplatin administration failed to protect mice from OIPN (Fig. 2D). To provide further evidence of a causal connection of duloxetine-mediated protection against OIPN with OCT2, we also evaluated the potential neuroprotective properties of duloxetine against the peripheral neurotoxicity associated with vincristine and paclitaxel, because these agents are not transported by OCT2 (25). These studies confirmed that OCT2 deficiency does not protect against vincristine- or paclitaxel-induced peripheral neurotoxicity, and that these toxicities were not prevented by pretreatment with duloxetine (Supplementary Fig. S5). Because no differences were observed in paw withdrawal force at baseline between groups (Supplementary Fig. S6), these findings provide further credence to our thesis that the ability of duloxetine to prevent against OIPN is dependent on inhibition of OCT2-mediated transport mechanisms.

### Duloxetine Affects OCT2 Biomarkers without Altering Oxaliplatin Plasma Levels

**FIGURE 3:**
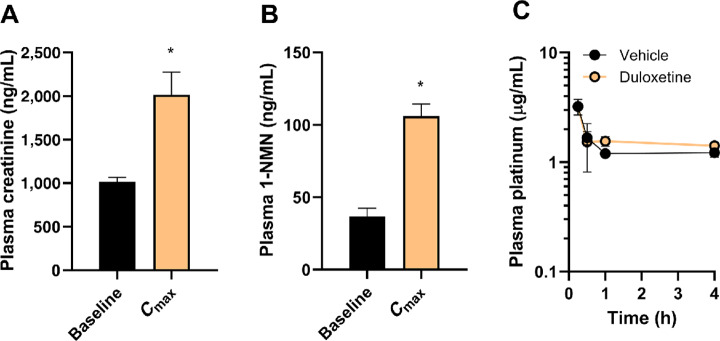
Duloxetine modulates OCT2 function *in vivo* without affecting plasma levels of oxaliplatin. Effect of duloxetine treatment on the plasma levels of the endogenous OCT2 biomarkers creatinine (**A**) and NMN (**B**) in wild-type mice. Bars represent level at baseline and the peak concentration (*C*_max_). **C,** Effect of duloxetine on the plasma concentration–time profile of oxaliplatin, measured as total platinum levels in wild-type mice. Bars and symbols represent mean ± SEM (*n* = 5 per group). *, *P* < 0.05 versus corresponding baseline levels.

To directly assess the influence of duloxetine on the function of OCT2 *in vivo*, levels of the endogenous OCT2 substrates creatinine (20) and NMN (21) were measured in plasma as inhibitor-sensitive biomarkers at baseline and following treatment with duloxetine. The observed maximal changes (∼2-fold) in the levels of creatinine (Fig. 3A) and NMN (Fig. 3B) in mice receiving a single dose of duloxetine were of the same order of magnitude as those reported previously in mice with an OCT2 deficiency (20, 21). Interestingly, the plasma pharmacokinetic profile of oxaliplatin was unchanged by pretreatment with a single dose of duloxetine in wild-type mice (Fig. 3C) and resulting average values for the AUC were similar between treatment groups (6.08 vs. 5.35 μg × hour/mL, respectively). These findings are congruent with the notion that the plasma levels of oxaliplatin are insensitive to genetic or pharmacologic knockout of OCT2 (5), and with our observation that duloxetine does not influence the function of OATP1B1/OATP1B3 and MATE1, transporters involved in the hepatic uptake and renal tubular secretion of oxaliplatin, respectively (21, 26).

### Duloxetine does not Reduce the Antitumor Efficacy of Oxaliplatin

**FIGURE 4:**
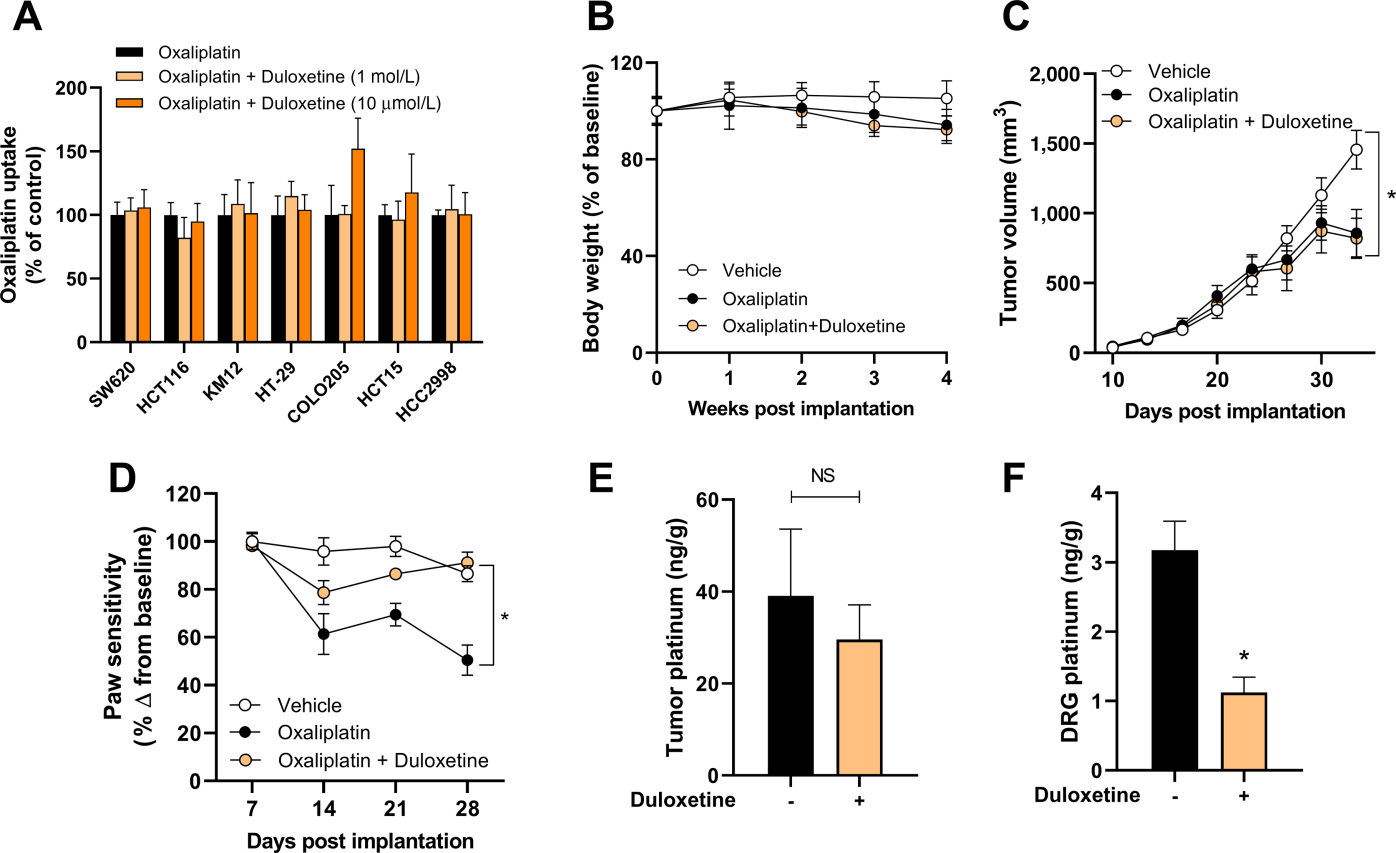
Duloxetine does not alter uptake and antitumor properties of oxaliplatin. **A,** Influence of duloxetine (1 or 10 μmol/L) on the intracellular accumulation of oxaliplatin in colorectal cancer cell lines *in vitro*. Influence of duloxetine pretreatment on oxaliplatin-related body weight loss (**B**), tumor volume of xenografted luciferase-expressing HCT116 cells (**C**), OIPN as measured by VFH test (**D**), levels of total platinum in tumor samples (**E**), and DRG neurons (**F**) after completion of a 3-week treatment regimen. Bars and symbols represent mean ± SD. Details of the treatment and analysis are described in the Materials and Methods section (*n* = 7–9). *, *P* < 0.05 versus vehicle control (**C**), versus oxaliplatin alone (**D**, **F**). NS, not statistically significant.

Although the addition of duloxetine to oxaliplatin-based regimens can prevent OIPN, experimental verification that such strategy does not compromise the anticancer efficacy of oxaliplatin is essential. Importantly, prior transcriptional profiling of drug transporters using RNA sequencing data (27) revealed that OCT2 is expressed at very low levels in human colorectal tumors and colorectal cancer cell lines compared to expression levels in DRG neurons (3). This expression signature is in line with our observation that duloxetine did not antagonize the cell-growth inhibition by oxaliplatin in a panel of seven colorectal cancer cell lines (Table 1; Supplementary Fig. S7). Moreover, duloxetine at concentrations as high as 10 μmol/L did not influence the cellular accumulation of oxaliplatin in any of these cell lines (Fig. 4A). These findings suggest that oxaliplatin is taken up into cancer cells independently of OCT2 and that this unknown mechanism is insensitive to duloxetine-mediated inhibition at physiologically relevant concentrations. The translational potential of a duloxetine-oxaliplatin combination therapy was further verified *in vivo* using mice xenografted with HCT116 colorectal cancer cells that were imaged using IVIS imaging to ensure equivalent tumor burden in each experimental group at the start of treatment (Supplementary Fig. S2). During a 3-week treatment period, the chosen regimen did not cause significant changes in body weight (Fig. 4B) and as predicted from the *in vitro* studies, duloxetine did not negatively influence the antitumor efficacy of oxaliplatin *in vivo* (Fig. 4C). In these tumor-bearing mice, duloxetine retained its ability to significantly improve the paw withdrawal sensitivity compared with animals receiving oxaliplatin alone (Fig. 4D). This provides further evidence for the thesis that duloxetine can effectively prevent the incidence and severity of both the acute and chronic forms of OIPN. As predicted from prior studies (3), oxaliplatin treatment in our models was not associated with detectable changes in nerve conduction amplitudes (Supplementary Fig. S8).

**TABLE 1:** Influence of duloxetine on oxaliplatin-mediated cell growth inhibition

IC_50_ (μmol/L)			
Cell line	Oxaliplatin	Oxaliplatin + Duloxetine (1 μmol/L)	Oxaliplatin + Duloxetine (10 μmol/L)
SW-620	1.40 ± 0.80	2.28 ± 1.02	3.32 ± 1.51
HCT-116	5.39 ± 2.63	5.40 ± 1.70	5.54 ± 3.60
COLO-205	2.95 ± 1.79	3.33 ± 2.44	N/A
HT-29	3.90 ± 1.00	4.24 ± 1.62	N/A
KM-12	>100	>100	N/A
HCC-2998	4.52 ± 2.15	6.25 ± 1.43	N/A
HCT-15	6.33 ± 2.04	6.35 ± 2.85	N/A

NOTE: Data represent mean ± SEM.

Abbreviations: IC_50_, concentration associated with 50% inhibition of cell growth; N/A, not available.

Following the completion of treatment in mice xenografted with HCT116 cells, tumor and DRG specimens were collected and analyzed for levels of total platinum originating from oxaliplatin. As anticipated, levels in tumors were similar between treatment groups (Fig. 4E), whereas levels in DRG neurons were significantly lower in animals that had received duloxetine (Fig. 4F). This observation supports the notion that duloxetine can restrict the entry of oxaliplatin specifically into DRG neurons and prevent OIPN without influencing access to tumor cells.

### Discussion

In the current study, we identified the organic cation transporter OCT2 as an important, previously unrecognized target of the FDA-approved antidepressant duloxetine in the peripheral nervous system. In particular, we found that duloxetine is a potent modulator of OCT2 activity, and that pretreatment of mice with this agent can diminish accumulation of the OCT2 substrate oxaliplatin in DRG neurons. In addition, we observed that pretreatment of mice with duloxetine can prevent OIPN, a side effect that is dependent on OCT2-mediated transport of oxaliplatin, without negatively affecting the plasma levels or antitumor efficacy of oxaliplatin.

OIPN is a debilitating and dose-limiting side effect that occurs in most patients receiving treatment with oxaliplatin and critically deteriorates quality of life. At present, no effective treatments are available to prevent OIPN, although several pharmacologic approaches have been proposed in the past decade (6), including strategies that are focused on inhibition of OCT2, a membrane transporter responsible for the uptake of oxaliplatin in DRG neurons (3). In a previously reported small-molecule library screen (5), we fortuitously identified duloxetine as a putative inhibitor of OCT2 function. Interestingly, several studies have shown that duloxetine has potential in the clinic to *treat* existing OIPN, although its utility in *preventing* this side effect remains unclear (28–31). The estimated pKa of duloxetine is 9.7, indicating that this compound will exist almost entirely in a cationic form at pH values between 5 and 9, and it has a hydrophobic-aromatic site separated by a short distance from the positive charge. The notion that these structural motifs are overrepresented in potent OCT2 inhibitors (32–34) is consistent with our current finding that duloxetine directly blocks the activity of OCT2 as well as some other but not all related organic cation transporters.

The mechanism by which duloxetine can affect chemotherapy-induced peripheral neurotoxicity has not been fully elucidated. It has been postulated that duloxetine increases the concentration of serotonin and norepinephrine in the postsynaptic region by inhibiting the reuptake of these neurotransmitters, and decreases the perception of pain (35, 36). In addition, duloxetine has a dose-dependent differential affinity for monoaminergic transporters, blocking the dopamine transporters at high concentrations, and thereby boosting synaptic dopamine availability (37). It is worth pointing out that structurally and pharmacologically related antidepressants, such as venlafaxine, are less effective than duloxetine in modulating neuropathic pain (38), and that venlafaxine was previously reported to not influence the OCT2-mediated transport of either metformin or the experimental probe substrate 1-methyl-4-phenylpyridinium (39). Similarly, the antiepileptic drugs gabapentin and pregabalin, which lack OCT2-inhibitory properties (IC_50_, >600 μmol/L; ref. 40), have failed in several preclinical models to significantly inhibit allodynia induced by oxaliplatin (41) and are not effectively preventing OIPN in randomized clinical trials (42). It is also noteworthy that duloxetine appears to be more effective in controlling neuropathic pain induced by oxaliplatin than by other chemotherapeutics, such as the tubulin poisons vincristine or paclitaxel (7, 43, 29), the neuronal transport of which is not dependent on OCT2 (17). This prior knowledge is consistent with our current findings on the relative lack of effect of duloxetine on vincristine- and paclitaxel-induced allodynia, regardless of OCT2-genotype status.

Interestingly, Kim and colleagues previously reported that intrathecal administration of the antihypertensive drugs phentolamine or prazosin prevented the antiallodynic action of duloxetine (44). The reversal of this phenotype was ascribed mechanistically to antagonism of spinal α1-adrenergic receptors based on the knowledge that both phentolamine and prazosin target this pathway. The current finding that duloxetine interacts with OCT2 provides an alternative explanation for the findings reported by Kim and colleagues in light of the fact that phentolamine (45) and prazosin (46) can potently inhibit mammalian organic cation transporters. Further studies are required to unravel the mechanistic details of this interaction and to further evaluate the thesis, supported by our preliminary *in vitro* studies, that the ability of duloxetine to ameliorate chemotherapy-induced peripheral neurotoxicity occurs independently of its own transport by OCT2.

On the basis of a VFH test to evaluate mechanical allodynia, we found that a single dose of duloxetine given before oxaliplatin is sufficient to offer complete protection against both acute and chronic forms OIPN in wild-type mice in a manner that resembles observations made in OCT1/2(−/−) mice. Compared with the parent drug, the major duloxetine metabolites 5-hydroxy-6-methoxy-duloxetine and 4-hydroxy-duloxetine-glucuronide (23) were found to display only weak OCT2-inhibitory properties, suggesting that the *in vivo* findings are likely mediated by duloxetine itself. The recorded nerve conduction amplitudes and velocities were not affected by oxaliplatin treatment in the applied murine model, and these measures were not further influenced by duloxetine pre-treatment. This observation is in line with recent studies indicating that oxaliplatin does not cause morphologic damage to DRG neurons in either wild-type mice or OCT1/2(−/−) mice, minimally affects the degeneration of caudal and sciatic nerve fibers, and lacks an effect on nerve conduction (3). These collective findings further support the notion that functional deficits in sensory transduction and neuronal firing of proprioceptors can continue to exacerbate behavioral outcomes despite the absence of tissue degeneration associated with OIPN (15).

Collectively, our study indicates that duloxetine can inhibit the function of OCT2 in DRG neurons, block the entry of the OCT2 substrate oxaliplatin into sites of injury within the peripheral nervous system, and thereby prevent acute and chronic forms of OIPN. The OCT2-targeting properties of duloxetine, combined with the lack of a pharmacokinetic drug-drug interaction and the absence of antagonism in models of colorectal cancer provide support for the further exploration of duloxetine as a therapeutic candidate for the prevention of OIPN.

## Supplementary Material

Supplementary Tables S1-S2, Figures S1-S8 Supplementary Table S1. Percentage of OCT2 inhibition by compounds reported to reduce platinum-induced toxicities. Supplementary Table S2. Validation of human, rat, and murine overexpressed cells by evaluating their ability to accumulate known prototypical transport substrates. Supplementary Figure S1. Chemical structure of duloxetine. Supplementary Figure S2. IVIS imaging of tumor bearing mice. Supplementary Figure S3. Metabolites of duloxetine do not inhibit OCT2 function. Supplementary Figure S4. Duloxetine is an OCT2 inhibitor that extensively binds to extracellular membrane. Supplementary Figure S5. Duloxetine does not prevent peripheral neurotoxicity associated with vincristine and paclitaxel. Supplementary Figure S6. Paw withdrawal force measured by VFH instrument before the start of the treatment in wild-type (WT) and OCT1/2(-/-) mice (n=5-10 per group). Supplementary Figure S7. Activity of oxaliplatin in various colorectal cancer cell lines. Supplementary Figure S8. Effect of duloxetine on sciatic and caudal nerve velocity and amplitude.
