# Supplementary material for: Targeting OCT2 with Duloxetine to Prevent Oxaliplatin-induced Peripheral Neurotoxicity
Source: Cancer Res Commun. 2022 Nov 3;2(11):1334–43. doi: 10.1158/2767-9764.CRC-22-0172 (PMC9730833; doi:10.1158/2767-9764.CRC-22-0172)
Supplement: Supplementary Tables S1-S2, Figures S1-S8 — Supplementary Table S1. Percentage of OCT2 inhibition by compounds reported to reduce platinum-induced toxicities. Supplementary Table S2. Validation of human, rat, and murine overexpressed cells by evaluating their ability to accumulate known prototypical transport substrates. Supplementary Figure S1. Chemical structure of duloxetine. Supplementary Figure S2. IVIS imaging of tumor bearing mice. Supplementary Figure S3. Metabolites of duloxetine do not inhibit OCT2 function. Supplementary Figure S4. Duloxetine is an OCT2 inhibitor that extensively binds to extracellular membrane. Supplementary Figure S5. Duloxetine does not prevent peripheral neurotoxicity associated with vincristine and paclitaxel. Supplementary Figure S6. Paw withdrawal force measured by VFH instrument before the start of the treatment in wild-type (WT) and OCT1/2(-/-) mice (n=5-10 per group). Supplementary Figure S7. Activity of oxaliplatin in various colorectal cancer cell lines. Supplementary Figure S8. Effect of duloxetine on sciatic and caudal nerve velocity and amplitude. [file crc-22-0172-s01.docx]

**Supplementary Materials**

**Targeting OCT2 with Duloxetine to Prevent Oxaliplatin-Induced Peripheral Neurotoxicity**

Mahesh R. Nepal^1,3^, Hanieh Taheri^1,3^, Yang Li^1,3^, Zahra Talebi^1^, Muhammad E. Uddin^1^, Yan Jin^1^, Duncan F. DiGiacomo^1^, Alice A. Gibson^1^, Maryam B. Lustberg^2^, Shuiying Hu^1,3^, and Alex Sparreboom^1^

**Authors’ Affiliations:** ^1^Division of Pharmaceutics and Pharmacology, College of Pharmacy & Comprehensive Cancer Center, The Ohio State University, Columbus, OH 43210; ^2^The Breast Center at Smilow Cancer Hospital at Yale, Yale School of Medicine, New Haven, CT 06511; and ^3^Division of Outcomes and Translational Sciences, College of Pharmacy & Comprehensive Cancer Center, The Ohio State University, Columbus, OH 43210.

**Supplementary Table S1.** Percentage of OCT2 inhibition by compounds reported to reduce platinum-induced toxicities.

| **Compound** | **% Inhibition** | **Observation** | **References** |
| --- | --- | --- | --- |
| Amitriptyline | 97.0 | Reduced oxaliplatin neuropathy in rats | Sada et al. 2012 (1) |
| Carvedilol | 92.3 | Reduced cisplatin nephrotoxicity in rats | Rodrigues et al. 2010 ([2](#_ENREF_49)) |
| Chlorpromazine | 98.9 | Reduced cisplatin nephrotoxicity | Ding, 1992 ([3](#_ENREF_50)) |
| Clomipramine | 87.5 | Reduced oxaliplatin neuropathy in rats | Ling et al. 2007 ([4](#_ENREF_51)) |
| Disopyramide | 73.3 | Reduced cisplatin nephrotoxicity in rats | Hanada et al. 1999 ([5](#_ENREF_52)) |
| **Duloxetine** | 85.7 | Reduced oxaliplatin neuropathy in humans | Yang et al. 2012 (6) |
| Ifenprodil | 84.6 | Reduced oxaliplatin neuropathy | Mihara et al. 2011 (7) |
| Parthenolide | 79.6 | Reduced cisplatin nephrotoxicity in rats | Francescato et al. 2007 (8) |
| Prochlorperazine | 98.5 | Reduced cisplatin nephrotoxicity in mice and rats | Kramer et al. 1989 ([9](#_ENREF_55)) |
| Purvalanol B | 94.2 | Reduced cisplatin nephrotoxicity in vitro and in vivo | Price et al. 2006 (10) |
| Pyrimethamine | 86.3 | Increased cisplatin nephrotoxicity in mice | Nakamura et al. 2010 (11) |
| Quercetin | 70.3 | Reduced cisplatin nephrotoxicity in vitro and in vivo | Kuhlmann et al. 1998 (12) |
| Rottlerin | 97.7 | Reduced cisplatin nephrotoxicity in vitro and in vivo | Basu et al. 1999 (13) |
| Trifluoperazine | 87.2 | Reduced oxaliplatin neurotoxicity | Shirahama et al. 2012 (14) |
| Tyrphostin | 84.2 | Reduced cisplatin nephrotoxicity | Novogrodsky et al. 1998 ([1](#_ENREF_61)5) |
| Verapamil | 82.2 | Reduced cisplatin nephrotoxicity in rats | Haragsim et al. 1992 (1[6](#_ENREF_62)) |

**Supplementary Table S2.** Validation of human, rat, and murine overexpressed cells by evaluating their ability to accumulate known prototypical transport substrates.

| **Transporter** | **Species** | **Substrate(s)** | **Fold-change** |
| --- | --- | --- | --- |
| OATP1B1 | Human | EβG | 7.28 ± 0.72 |
| OATP1B2 | Mouse | EβG | 24.1 ± 7.38 |
| OATP1B3 | Human | CCK-8 | 5.42 ± 1.43 |
| OCT1 | Human | TEA | 112 ± 34.9 |
| OCT2 | Human | ASP | 2.93 ± 0.07 |
|  |  | TEA | 62.8 ± 5.54 |
|  |  | Oxaliplatin | 5.38 ± 0.46 |
| OCT2 | Mouse | ASP | 6.70 ± 0.70 |
|  |  | TEA | 63.1 ± 3.63 |
|  |  | Oxaliplatin | 3.43 ± 0.09 |
| OCT3 | Human | Metformin | 18.9 ± 6.84 |
| OCT2 | Rat | TEA | 98.1 ± 13.3 |
| MATE1 | Human | TEA | 5.56 ± 0.19 |
|  | Mouse | TEA | 7.32 ± 4.28 |

*Abbreviations:* EβG, estradiol [6,7-3H(N)]-17β-D-glucuronide; CCK-8 cholecystokinin octapeptide [proprionyl-3H(N)]-CCK-8 TEA, [ethyl-1-14C]-tetraetylammonium chloride; ASP, 4-(4-(dimethylamino)styryl)-N-methylpyridinium iodide. Data are expressed as the mean ± SEM of fold differences between uptake kinetics (pmol/min/mg protein) of radiolabeled probe substrates in cells overexpressing the transporter of interest and corresponding control cells transfected with an empty control vector.


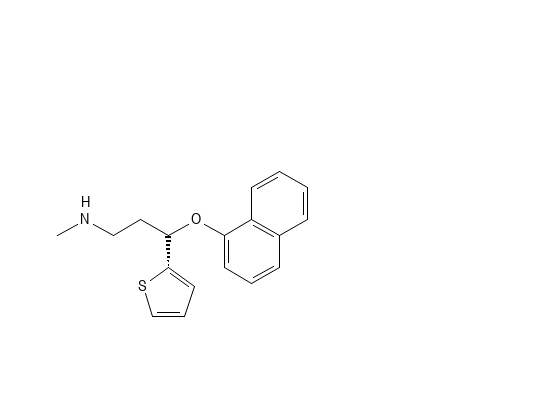


**Supplementary Figure S1.** Chemical structure of duloxetine.

**Control**

**Oxaliplatin**

**Oxaliplatin + Duloxetine**


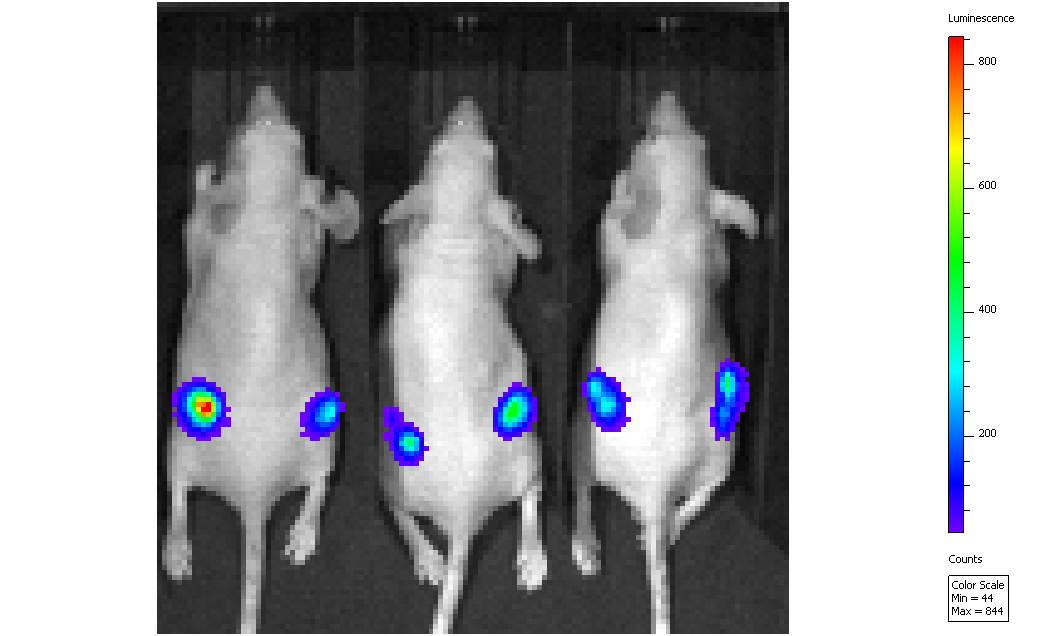

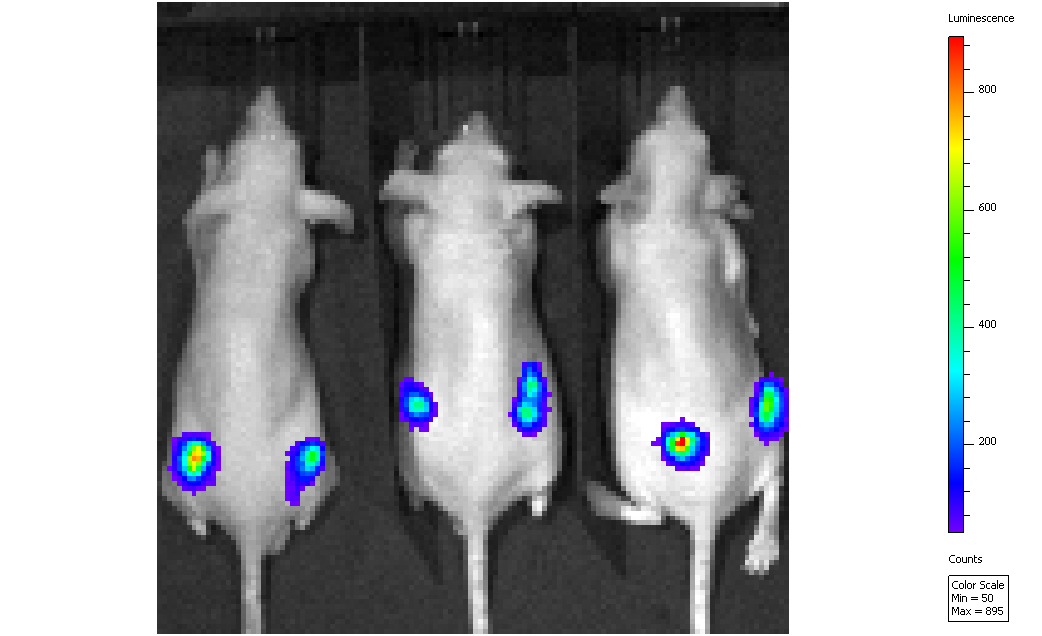

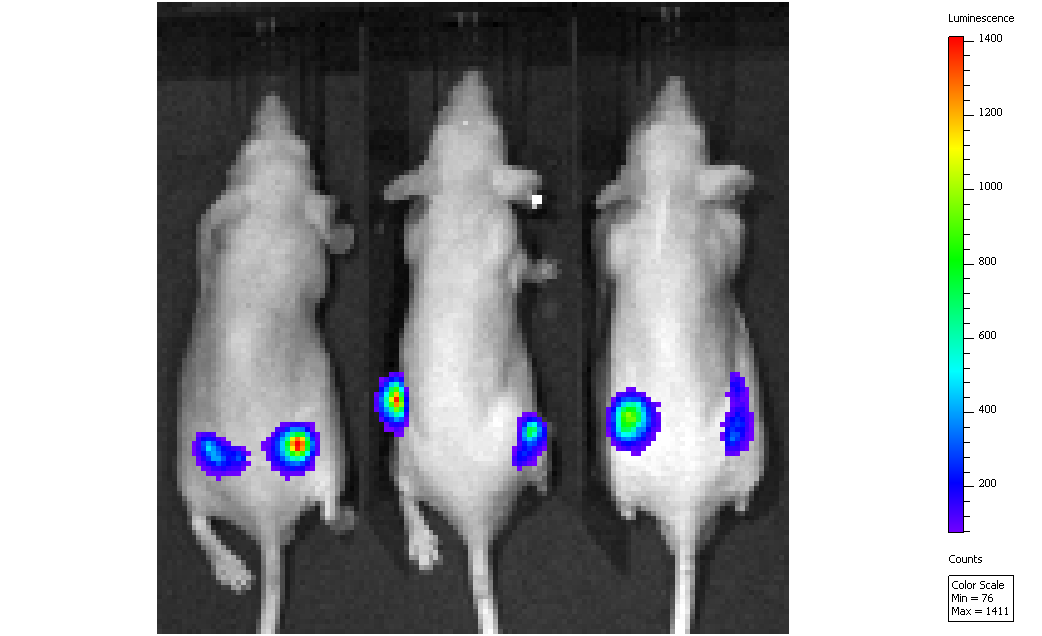


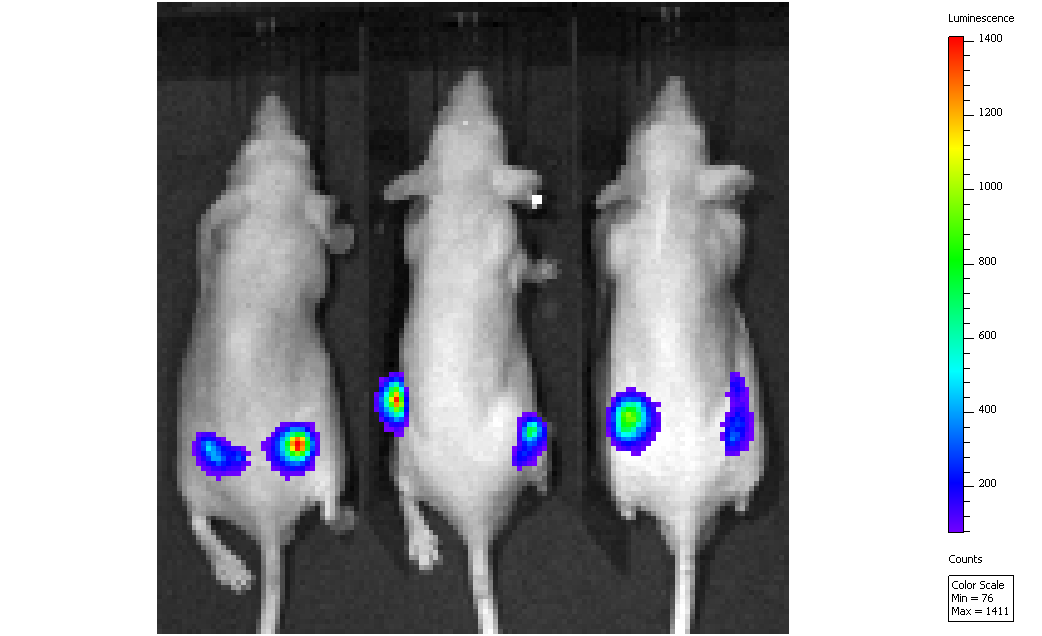


**Supplementary Figure S2.** IVIS imaging of tumor bearing mice. Mice injected with tumor cells containing luciferase activities were treated with D-luciferin intraperitoneally and IVIS imaging was carried out after 10 min using a 1-s exposure time. Top panel, representative imaging of baseline tumor burden. Bottom panel, mean ± SEM of imaging from baseline tumor burden.


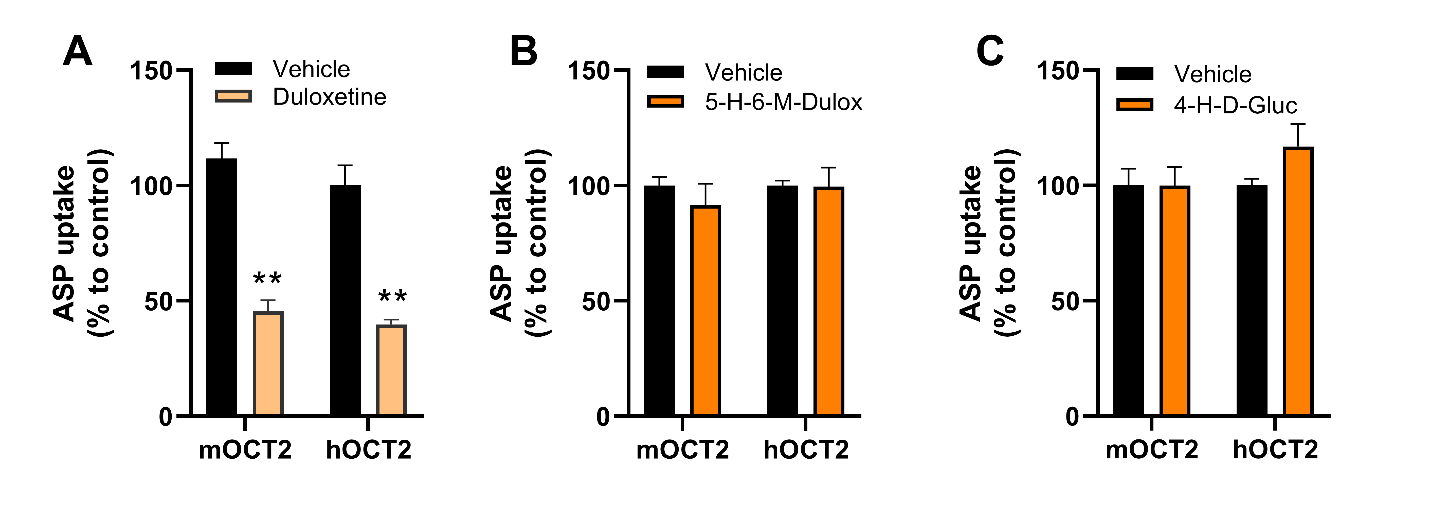


**Supplementary Figure S3.** Metabolites of duloxetine do not inhibit OCT2 function. HEK293 cells overexpressing mouse OCT2 or human OCT2 were pre-treated with either duloxetine (**A**) or its metabolites 5-hydroxy-6-methoxy-duloxetine (5-H-6-M-Dulox) (**B**) and 4-hydroxy-duloxetine-glucuronide (4-H-D-Gluc) (**C**) and transport function was evaluated by measuring uptake of ASP. Bars represent mean ± SEM. ***P*<0.01 vs vehicle.


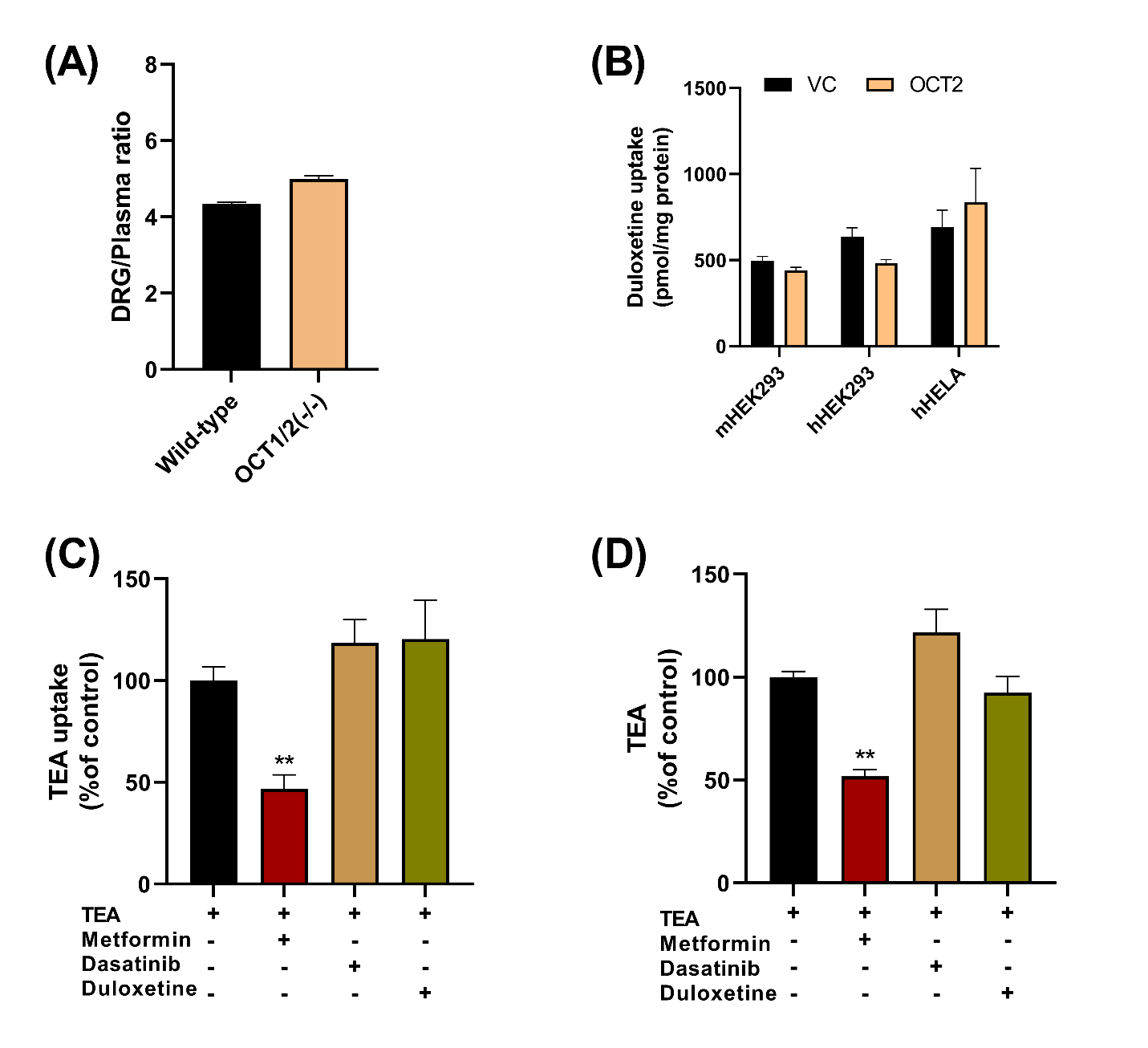


**Supplementary Figure S4.** Duloxetine is an OCT2 inhibitor that extensively binds to extracellular membrane. **(A)** DRG to plasma ratio of duloxetine in wild-type and OCT1/2(-/-) mice following intravenous injection of 20 mg/kg radiolabeled duloxetine (n=5 in each group). **(B)** Influence of duloxetine on human HELA OCT2 and human and mouse HEK293-overexpressed and vector control (VC) cells (n= 3 in each group). **(C-D)** Competitive counter flow assay of TEA with duloxetine as a test substance. Human HELA OCT2 **(C)** and mouse HEK293 OCT2 **(D)**-overexpressed cells were preloaded with 2 µM TEA for 3 min and TEA with or without duloxetine was added in the mixture and further incubated for another one min. Metformin and dasatinib were used as a positive control and negative controls, respectively. Each bar represents mean ± SEM of two independent experiments (n=6). ***P*<0.01 vs vehicle.


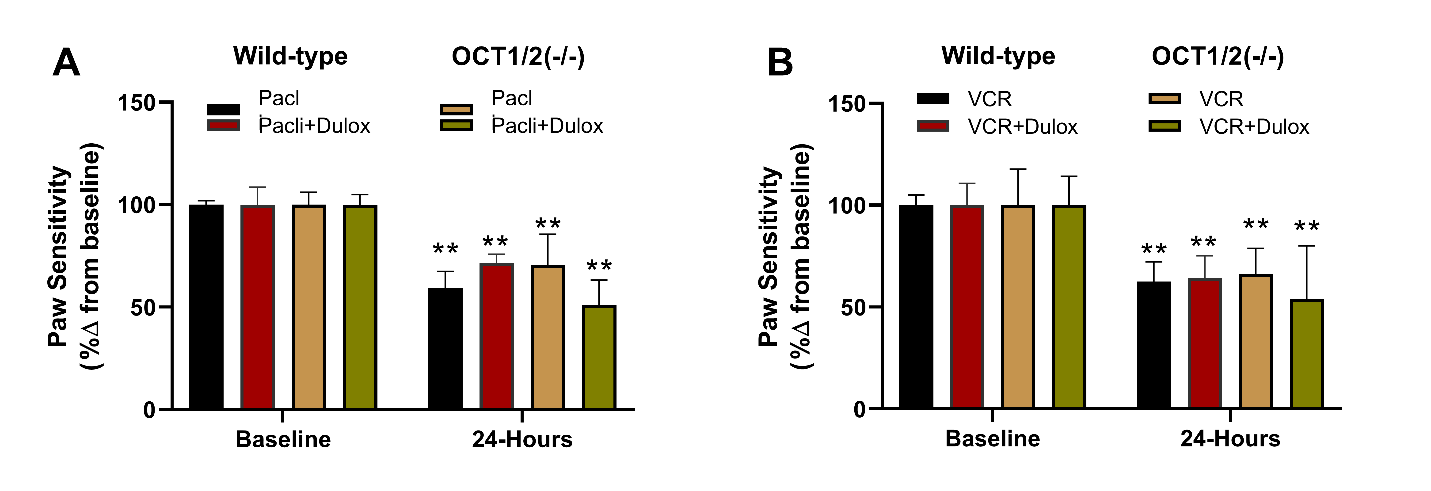


**Supplementary Figure S5.** Duloxetine does not prevent peripheral neurotoxicity associated with vincristine and paclitaxel. Mechanical allodynia in mice was measured by Von Frey Hair test at baseline and 24 h following treatment with either paclitaxel (Pacli) (**A**) or vincristine (VCR) (**B**) in wild-type (WT) and OCT1/2(-/-) mice(n=6). Duloxetine (Dulox) was administered 1 h prior to vincristine or paclitaxel treatment. ***P*<0.01 vs baseline.


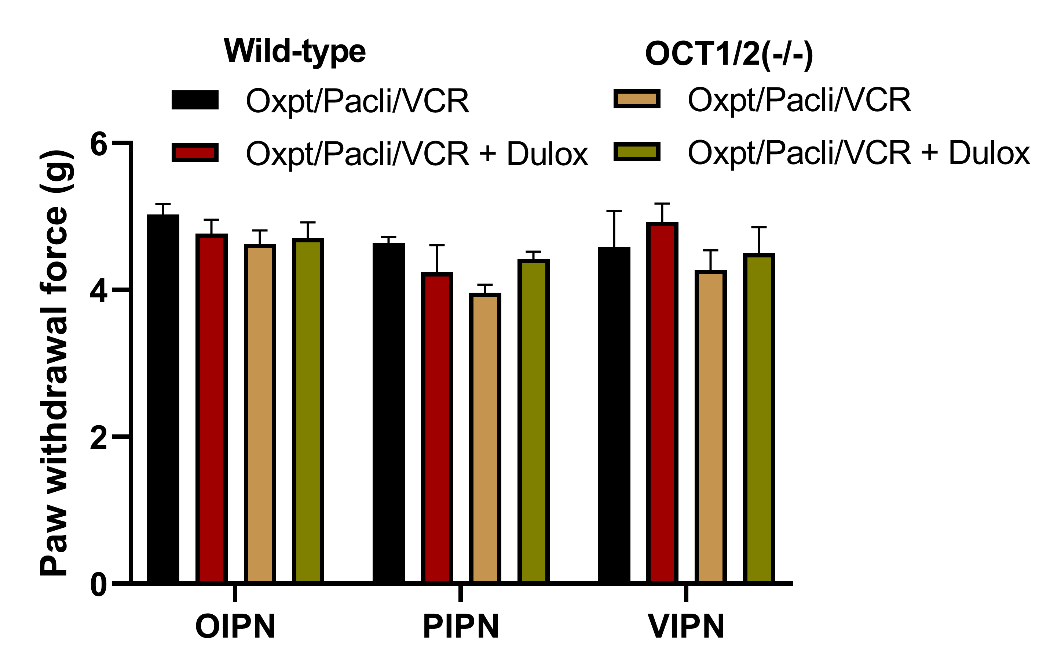


**Supplementary Figure S6.** Paw withdrawal force measured by VFH instrument before the start of the treatment in wild-type (WT) and OCT1/2(-/-) mice (n=5-10 per group). Oxpt/Pacli/VCR represents oxaliplatin, paclitaxel and vincristine, respectively. No statistically significant difference was noted between groups.


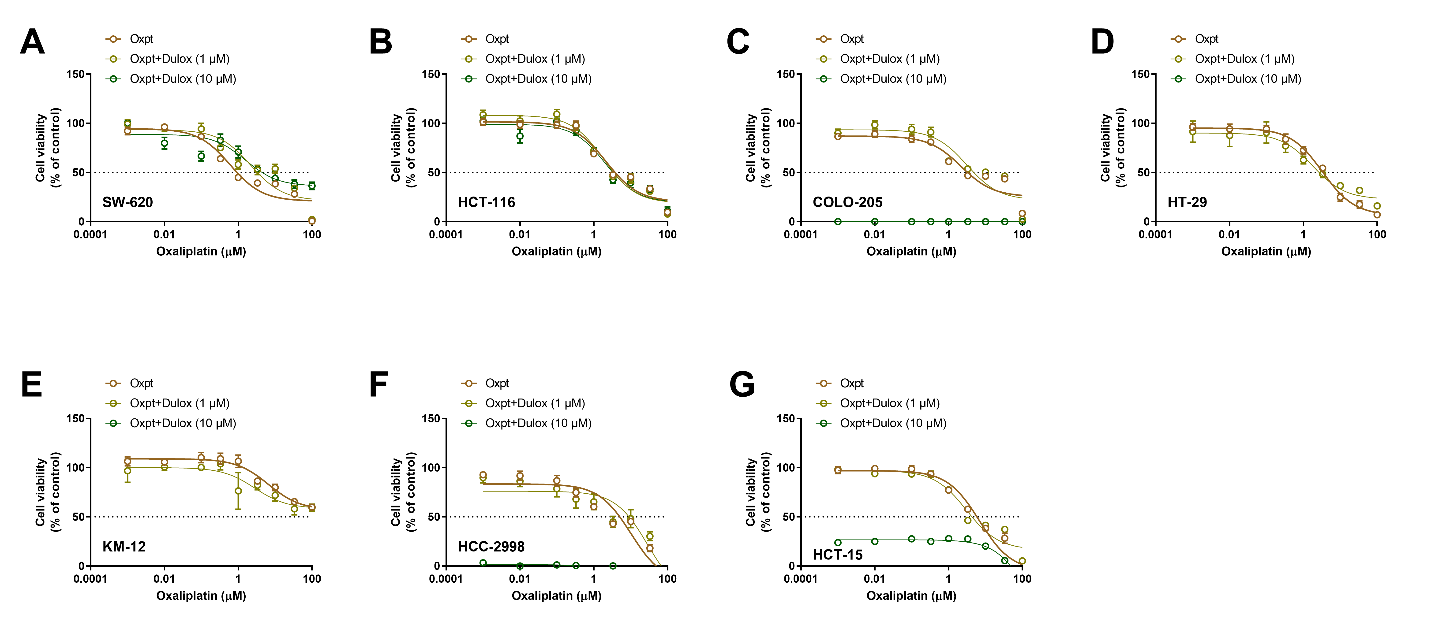


**Supplementary Figure S7.** Activity of oxaliplatin in various colorectal cancer cell lines. Cell viability was conducted using MTT assay following continuous exposure up to 72-hours to either oxaliplatin alone or combination of oxaliplatin with duloxetine at either 1 µM or 10 µM. Sigmoidal curves were generated using a nonlinear regression curve fit. Oxpt, Oxaliplatin; Oxpt+dulox, combination of oxaliplatin and duloxetine. Oxaliplatin combined with 10 µM duloxetine in HT-29 and KM-12 cells generated negative values, thus, not shown in the figure (panel D and E).


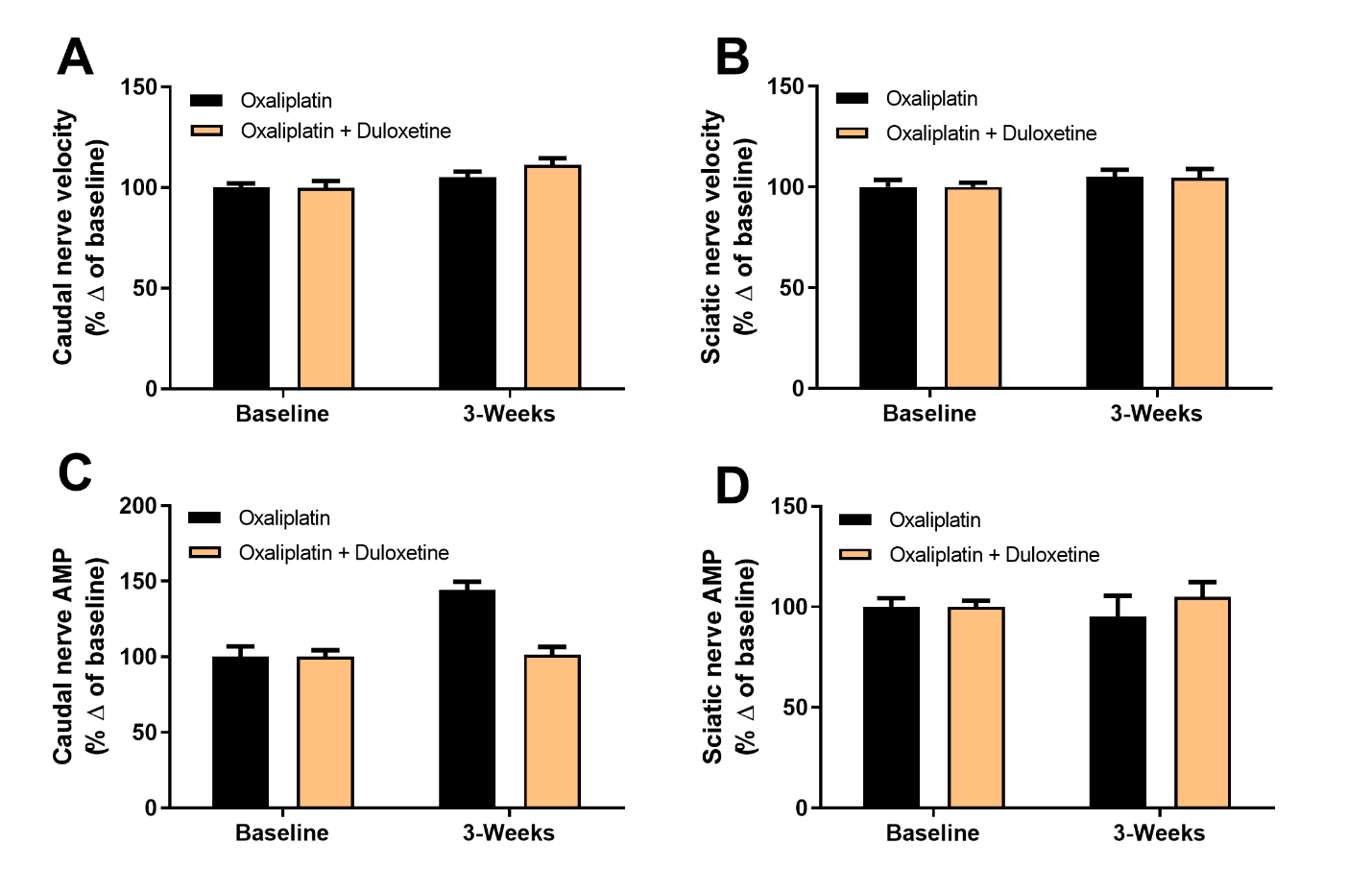


**Supplementary Figure S8.** Effect of duloxetine on sciatic and caudal nerve velocity and amplitude. HCT116 xenograft mice treated with oxaliplatin, or duloxetine followed by oxaliplatin were subjected to measurement of sciatic and caudal nerve velocity and amplitude at baseline (before treatment) and at the end of the study period. Data represent percentage changes from baseline values.

**Supplementary references**

1. Sada H, Egashira N, Ushio S, Kawashiri T, Shirahama M, Oishi R. Repeated administration of amitriptyline reduces oxaliplatin-induced mechanical allodynia in rats. *J Pharmacol Sci* 2012;118(4):547-51.

2. Rodrigues MA, Rodrigues JL, Martins NM, Barbosa F, Curti C, Santos NA*, et al.* Carvedilol protects against the renal mitochondrial toxicity induced by cisplatin in rats. *Mitochondrion* 2010;10(1):46-53.

3. Ding DC. Clinical multivariate statistical analysis of nephrotoxicity induced by cisplatin. C*hin. J. Oncol.* 1992;14(1):64-6.

4. Ling B, Authier N, Balayssac D, Eschalier A, Coudore F. Behavioral and pharmacological description of oxaliplatin-induced painful neuropathy in rat. *Pain* 2007;128(3):225-34.

5. Hanada K, Odaka K, Kudo A, Ogata H. Effects of disopyramide and verapamil on renal disposition and nephrotoxicity of cisplatin in rats. *Pharm Res* 1999;16(10):1589-95.

6. Yang YH, Lin JK, Chen WS, Lin TC, Yang SH, Jiang JK*, et al.* Duloxetine improves oxaliplatin-induced neuropathy in patients with colorectal cancer: an open-label pilot study. *Support Care Cancer* 2012;20(7):1491-7.

7. Mihara Y, Egashira N, Sada H, Kawashiri T, Ushio S, Yano T*, et al.* Involvement of spinal NR2B-containing NMDA receptors in oxaliplatin-induced mechanical allodynia in rats. *Mol Pain* 2011;7:8.

8. Francescato HD, Costa RS, Scavone C, Coimbra TM. Parthenolide reduces cisplatin-induced renal damage. *Toxicology* 2007;230(1):64-75.

9. Kramer RA. Protection against cisplatin nephrotoxicity by prochlorperazine. *Cancer chemotherapy and pharmacology* 1989;25(3):156-60.

10. Price PM, Yu F, Kaldis P, Aleem E, Nowak G, Safirstein RL*, et al.* Dependence of cisplatin-induced cell death in vitro and in vivo on cyclin-dependent kinase 2. *J Am Soc Nephrol* 2006;17(9):2434-42.

11. Nakamura T, Yonezawa A, Hashimoto S, Katsura T, Inui K. Disruption of multidrug and toxin extrusion MATE1 potentiates cisplatin-induced nephrotoxicity. *Biochem Pharmacol* 2010;80(11):1762-7.

12. Kuhlmann MK, Horsch E, Burkhardt G, Wagner M, Köhler H. Reduction of cisplatin toxicity in cultured renal tubular cells by the bioflavonoid quercetin. *Arch Toxicol* 1998;72(8):536-40.

13. Basu A, Akkaraju GR. Regulation of caspase activation and cis-diamminedichloroplatinum(II)-induced cell death by protein kinase C. *Biochemistry* 1999;38(14):4245-51.

14. Shirahama M, Ushio S, Egashira N, Yamamoto S, Sada H, Masuguchi K*, et al.* Inhibition of Ca2+/calmodulin-dependent protein kinase II reverses oxaliplatin-induced mechanical allodynia in rats. *Mol Pain* 2012;8:26.

15. Novogrodsky A, Weisspapir M, Patya M, Meshorer A, Vanichkin A. Tyrphostin 4-nitrobenzylidene malononitrile reduces chemotherapy toxicity without impairing efficacy. *Cancer Res* 1998;58(11):2397-403.

16. Haragsim L, Zima T. Protective effects of verapamil on cis-platinum and carboplatinum nephrotoxicity in dehydrated and normohydrated rats. *Biochem Int* 1992;28(2):273-6.
